# Nanochelate-based BCc1 delivery and its impact on key regulatory pathways in BALB/c breast cancer: An analysis of *Beclin-1*, *ATG-4B*, *ATG-7*, and *mTOR* expression

**DOI:** 10.1016/j.bbrep.2025.102418

**Published:** 2026-01-06

**Authors:** Fereshteh Moheb Afzali, Masoumeh Heshmati, Ali Salimi, Somayeh Kalanaky, Saideh Fakharzadeh, Maryam Hafizi, Mohammad Esmail Akbari, Mohammad Hassan Nazaran, Mehrdad Hashemi

**Affiliations:** a Department of Cellular and Molecular Biology, TeMS.C., Islamic Azad University, Tehran, Iran; b Cellular and Molecular Research Center, Iran University of Medical Sciences, Tehran, Iran; c Department of Research and Development, Sodour Ahrar Shargh Company, Tehran, Iran; d Cancer Research Center, Shahid Beheshti University of Medical Science, Tehran, Iran; e Farhikhtegan Medical Convergence Sciences Research Center, Farhikhtegan Hospital., Faculty of Medicine., TeMS.C., Islamic Azad University, Tehran, Iran; f Department of Genetics, Faculty of Medicine, TeMS.C., Islamic Azad University, Tehran, Iran

**Keywords:** Breast cancer, BCc1 nanomedicine, Nanochelating technology, Autophagy

## Abstract

**Background:**

Breast cancer (BC) ranks as the most prevalent cancer type among women globally. Nanoparticle technology, a promising approach, plays a crucial role in effective cancer diagnosis and treatment. In this context, researchers investigated the efficacy of BCc1 nanomedicine, which utilizes nanochelating technology and possesses anti-neoplastic properties, in mice with breast tumors. Notably, this study represents the first global exploration of BCc1 nanomedicine's potential to induce autophagy, a process mediated by autophagy-related genes (*Beclin-1*, *ATG-4B*, *ATG-7*, and *mTOR*), while evaluating tumor cell death.

**Methods:**

In this study, female BALB/c mice bearing 4T1 mammary tumors received daily treatments with BCc1 nanomedicine for 24 consecutive days via two administration routes: intraperitoneal (i.p.) injection and oral administration by gavage. The research investigated the impact of BCc1 nanomedicine on autophagy induction. Importantly, BCc1 nanomedicine played a role in mitigating tumor cell death severity by activating essential genes. Real-time PCR facilitated detailed gene expression analysis during the 24-day treatment period.

**Results:**

Cyclophosphamide and BCc1 nanomedicine exhibited distinct regulatory effects on autophagy-associated genes. *Beclin-1* expression was significantly upregulated in both cyclophosphamide-treated and BCc1-administered groups compared to controls. In BCc1-treated mice, *ATG-4B* and *ATG-7*—genes essential for autophagosome formation and maturation—were markedly downregulated across all dosing regimens. Concurrently, BCc1 induced a significant reduction in *mTOR* expression, consistent with the removal of a major inhibitory checkpoint in autophagy initiation. Taken together, these findings suggest that BCc1 exerts a stage-specific influence on autophagy, potentially enhancing its initiation phase while attenuating subsequent maturation steps.

**Conclusion:**

In summary, BCc1 nanomedicine demonstrates therapeutic potential in BC, in part through the modulation of autophagy pathways. The observed gene expression profile—characterized by *mTOR* suppression and *Beclin-1* upregulation alongside reduced *ATG-4B* and *ATG-7* expression—indicates a selective enhancement of autophagy initiation, coupled with alterations in autophagosome maturation. This nuanced modulation of autophagy may contribute to BCc1's anti-tumor activity and warrants further investigation into its stage-specific mechanistic effects in cancer therapy.

## Highlights

• BCc1 nanomedicine demonstrates therapeutic potential in breast cancer.
• The efficacy of BCc1 nanomedicine possesses *anti*-neoplasmic properties, in mice with breast tumors.
• BCc1 nanomedicine's capacity to induce autophagy.
• Enhancement of autophagy initiation characterized by mTOR suppression and Beclin-1 upregulation alongside reduced ATG-4B and ATG-7 expression.

## Graphical abstract

**Figure:**
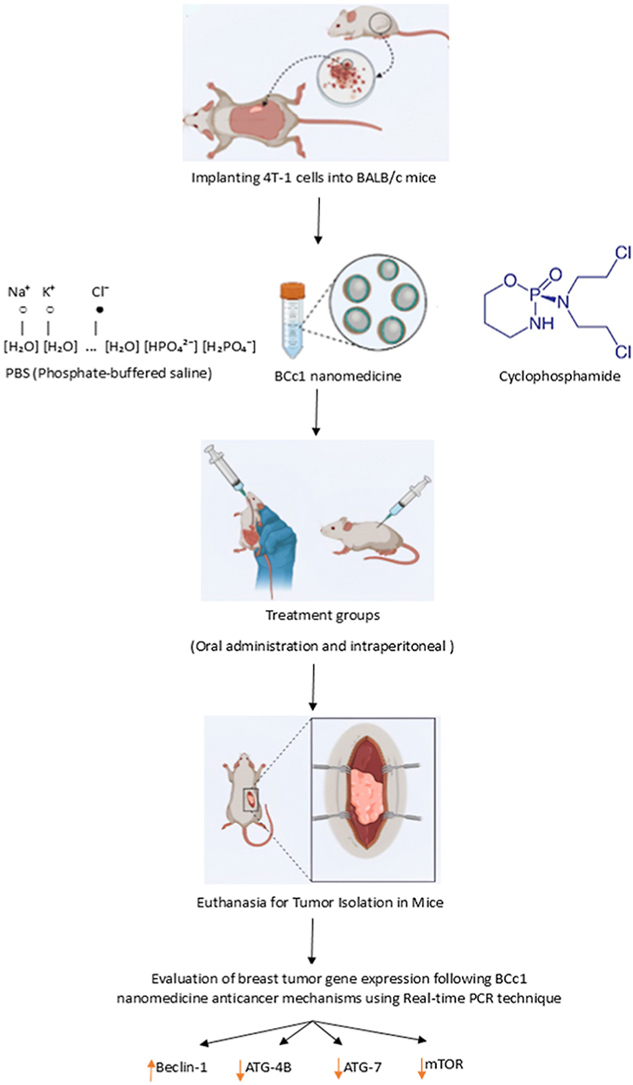

## 1 Introduction

Breast cancer (BC) remains the most frequently diagnosed malignancy in women worldwide, posing ongoing clinical challenges despite advances in early detection and treatment [1]. While genetic mutations play a pivotal role in tumor initiation, emerging evidence underscores the importance of epigenetic alterations in sustaining aberrant gene expression programs that drive tumor progression and therapeutic resistance [2,3].

Conventional interventions, including surgery, chemotherapy, radiotherapy, and hormone therapy, often lack tumor specificity, leading to systemic toxicity and limited efficacy [4]. Nanomedicine has emerged as a transformative approach in oncology, offering enhanced delivery precision, reduced off-target effects, and improved intratumoral drug accumulation through engineered nanoparticles [5,6].

Among these, nanochelating platforms, designed to sequester essential metal ions, provide a dual therapeutic strategy by combining targeted delivery with disruption of metal-dependent oncogenic pathways [7]. The integration of tumor-specific ligands further enhances selectivity and minimizes systemic toxicity [8].

Iron chelation, in particular, targets the elevated iron demand of rapidly proliferating cancer cells, offering a mechanistic rationale for its use in BC therapy [9,10].

Autophagy and apoptosis are interlinked cellular processes with context-dependent roles in cancer progression and treatment response [11,12]. Autophagy can function as a cytoprotective mechanism under metabolic stress, yet under certain conditions it promotes cell death, depending on tumor type and microenvironmental factors [13]. The dynamic interplay between autophagic and apoptotic pathways represents a promising therapeutic axis for intervention in BC [12,14].

BCc1 nanomedicine, developed using proprietary nanochelating technology, exhibits distinct physicochemical and biological features [15]. Constructed on a synthetic organic scaffold, BCc1 demonstrates minimal cytotoxicity under oxidative stress, selectively targets BC stem-like cells, and induces G1-phase cell cycle arrest while modulating redox balance and immune signaling pathways [15].

Preclinical studies show that BCc1 enhances T-cell activity, modulates pro- and anti-inflammatory cytokine profiles, suppresses metastatic dissemination, and prolongs survival in murine BC models [15,16]. Collectively, these results position BCc1 as a compelling therapeutic agent that combines selective cytotoxicity with immunomodulatory and antimetastatic capabilities [15,16]. Early clinical trials in gastric cancer further support its translational potential [17,18].

Despite these advances, the influence of BCc1 on autophagy-related gene expression in the context of BC immunotherapy remains poorly defined. Given the critical role of autophagic regulation in controlling tumor immune evasion and cell death, the present study investigates the effects of BCc1 on key autophagy-associated genes *ATG-4B*, *ATG-7*, *Beclin-1* and the *mTOR* signaling pathway using a BALB/c mouse model of BC. These genes are central to autophagosome initiation, maturation, and regulation through nutrient-sensing and stress-response mechanisms. By elucidating BCc1's impact on autophagy signaling and downstream antitumor responses, this work aims to clarify its potential as a novel immunotherapeutic agent in BC.

## 2 Materials and methods

### 2.1 Culturing the 4T-1 cell line

The 4T-1 cell line (ATCC CRL-2539) was sourced from the Pasteur Institute of Iran's cell bank. Mouse BC cells underwent maintenance in RPMI-1640 culture medium (INOCLON, Iran), with the addition of 10 % heat-treated fetal bovine serum (FBS; Gibco, Germany) to support cellular growth and viability, as well as antibiotics (100U/ml penicillin and 100μg/ml streptomycin; INOCLON, Iran), 4 mM l-glutamine, 1 mM sodium pyruvate, and 50 μM 2-mercaptoethanol. The cultures were maintained under standard incubator conditions at 37 °C with 5 % CO_2_ and 95 % relative humidity.

### 2.2 Percentage of living cells

Trypan blue solution (0.4 % in normal saline) was mixed with the cell suspension at a 1:5 ratio. After incubating for 1–2 min, the number of viable (unstained) and non-viable (stained) cells was determined using a hemocytometer. The total number of live cells was calculated, and cells were subsequently resuspended in PBS at a concentration of 5 × 10^4^ cells/ml for further experiments.

### 2.3 Animal model and housing conditions

Seventy female BALB/c mice (6–8 weeks old; 20 ± 0.5 g) were procured from the Pasteur Institute of Iran (Karaj) and housed under specific pathogen-free (SPF) conditions in standard cages. Following a one-week acclimatization period, the animals were maintained with ad libitum access to food and water in a temperature-controlled environment (20–22 °C) under a 12:12 h light/dark cycle.

All experimental procedures were conducted in strict accordance with the principles of the 3Rs (Replacement, Reduction, Refinement) and reported in compliance with ARRIVE 2.0 guidelines, ensuring transparency in sample size justification, ethical review, and implementation of humane endpoints.

Animals were closely monitored at least once daily during acclimatization and early experimental phases, increasing to twice daily assessments during periods of active tumor progression. Humane endpoints were rigorously defined and included tumor volumes exceeding 2000 mm^3^, presence of ulceration, body weight loss greater than 20 %, or signs of impaired mobility or lethargy, with immediate euthanasia performed upon reaching any of these criteria to minimize suffering.

### 2.4 Strain and sex selection criteria

Female BALB/c mice were selected owing to their well-established susceptibility to BC models, a trait shaped by strain-specific hormonal milieu, immune responsiveness, and heightened sensitivity to tumor induction. The choice also accounted for the Lee–Boot effect, recognizing its potential influence on immune–endocrine interactions relevant to cancer progression. As a genetically inbred strain, BALB/c mice exhibit minimal inter-individual immunological variability, thereby enhancing experimental reproducibility and enabling precise statistical comparisons while reducing the number of animals required to achieve robust power.

### 2.5 Ethical approval

All animal experiments were carried out in strict adherence to both institutional and national ethical regulations and were formally approved by the Institutional Animal Ethics Committee of the Islamic Azad University of Tehran Medical Sciences (Approval code: IR.IAU.PS.REC.1400.341). The committee, composed of veterinary experts and independent scientific reviewers, thoroughly evaluated the complete experimental protocol, including procedures such as tumor fragment implantation, subcutaneous inoculation of tumor cells, anesthesia, surgical interventions, and humane euthanasia. All procedures were performed under continuous veterinary oversight, ensuring compliance with established animal welfare guidelines and the highest standards of ethical conduct. The official approval document is publicly accessible online at: https://ethics.research.ac.ir/IR.IAU.PS.REC.1400.341.

Animals were randomly assigned to experimental groups using a computer-generated randomization list to minimize selection bias. Investigators responsible for data collection and analysis were blinded to group allocation throughout the study. Humane endpoints were predefined, and animals were monitored at least once daily for general health status, behavior, body weight, food and water intake, and clinical signs of pain or distress by trained personnel. Any animal exhibiting signs of undue suffering was promptly evaluated by a veterinarian.

Euthanasia was performed humanely using an overdose of sodium pentobarbital (≥100 mg/kg, intraperitoneal injection) as the single approved method. Although the original protocol proposed cervical dislocation or the “spinal method” as secondary options, the ethics committee approved the exclusive use of sodium pentobarbital to ensure maximal animal welfare. No deviations from this approved procedure occurred. Additionally, the committee determined that a group size of n = 10 per experimental arm was sufficient to achieve both statistical validity and ethical acceptability. All procedures adhered to the principles of the 3Rs (Replacement, Reduction, and Refinement) to minimize animal suffering and optimize scientific integrity.

### 2.6 Tumor cell injection

A suspension of ($1x10^{6}\;cells/ml$) in sterile PBS solution was prepared and administered subcutaneously into five sites of BALB/c mice. The mice were monitored weekly to assess tumor growth. Upon reaching a tumor diameter of 20 mm in tumor-bearing mice, the tumors were excised and utilized for modeling.

### 2.7 Tumor implants in groups of mice

Mice were anaesthetized using ketamine/xylazine (Zist Royesh, Iran). The tumor pieces were then surgically transplanted to the left side of the mice; The mice were kept in a cage in a room maintained at 25 °C following surgery for recuperation.

### 2.8 BCc1 nanomedicine: Formulation and physicochemical characterization

BCc1 Nanomedicine is a self-assembled iron-chelating nanoagent developed via proprietary nanoplate synthesis technology (US Patent No. 8288587B2) [19]. The nanocomplex has a defined particle size range of 45–47 nm and is engineered for enhanced stability and targeted delivery [16].

### 2.9 Preclinical toxicity evaluation and dose selection procedures

Toxicological studies confirm BCc1's low systemic toxicity, with an LD_50_ exceeding 109 mg/kg. Notably, administration does not negatively impact circulating erythrocyte levels, suggesting hematologic safety [20,21]. The therapeutic dose used in this study was selected based on FDA-recommended preclinical extrapolation guidelines and corroborated by prior in vivo safety data indicating a no-observed-adverse-effect level (NOAEL) at the selected dose [16,22].

### 2.10 Experimental assays for mechanistic and antitumor activity assessment

BCc1 has demonstrated robust antitumor activity in both in vitro and in vivo models, particularly in gastric cancer. Mechanistically, its iron-chelating function disrupts cellular iron homeostasis, thereby promoting oxidative stress and reactive radical generation. These molecular events contribute to downstream signaling activation and enhanced immunogenic cell death [16].

### 2.11 Dynamic light scattering (DLS) analysis

The hydrodynamic size profile of BCc1 nanoparticles was determined using dynamic light scattering (DLS). Measurements were carried out with a Malvern Nano ZS instrument (ZEN360, red badge series) at the KEFA Laboratory, Sharif University of Technology. Prior to analysis, BCc1 samples were dispersed in deionized water and subjected to brief sonication to minimize the formation of aggregates. The resulting suspension was transferred to a clean quartz cuvette, equilibrated to room temperature, and analyzed following the manufacturer's standard operating protocol.

### 2.12 Reference stability characteristics (published data)

The acid–stability profile of BCc1 has been previously characterized using pH-adjusted aqueous systems ranging from pH 2.0 to 6.0 and evaluated by spectroscopic analyses following incubation at 37 °C [23]. These published data demonstrate that BCc1 retains approximately 99 % of its structural integrity under acidic conditions, with no requirement for stabilizing excipients. In the present study, these validated findings were used solely to substantiate the rationale for the selected administration route.

### 2.13 Reference aqueous solubility characteristics (published data)

The solubility behavior of BCc1 under physiologic and acidic pH conditions has been reported previously [19,23]. BCc1 exhibits complete solubility and remains uniformly dispersed across pH 2.0–7.4. These established properties were referenced in the current work to support justification of the formulation and administration strategy. No solubility assays were performed as part of the present animal study.

### **2.14** Animal models and experimental procedures

Seventy female BALB/c mice were randomized into seven groups (n = 10 per group) based on a comprehensive sample size determination strategy. Tumor stocks were established by subcutaneous injection of 4T-1 breast carcinoma cells into donor mice. Upon tumor maturation, donor animals were humanely euthanized, and tumors were aseptically excised, sectioned into 2–3 mm fragments, and stored in sterile saline supplemented with antibiotics until implantation. Recipient mice were anaesthetized with ketamine (100 mg/kg) and xylazine (10 mg/kg) administered intraperitoneally.

Preemptive analgesia was provided through subcutaneous administration of buprenorphine (0.05 mg/kg) and meloxicam (2 mg/kg) 30 min prior to incision. Tumor fragments containing approximately $1x10^{6}\;cells/ml$ were implanted into the flank of recipients, with incisions closed using surgical adhesive and staples.

Postoperative analgesia was maintained with buprenorphine (0.05 mg/kg SC) every 8–12 h for 48 h and meloxicam (2 mg/kg SC) once daily for 2–3 days. When warranted and approved by veterinary staff, sustained-release buprenorphine formulations were employed to provide extended analgesia per institutional protocols.

Post-surgical recovery involved placement on heated pads, provision of softened food and hydrogel, and close monitoring for pain, wound healing, and general well-being, with handling minimized during the immediate postoperative period to reduce stress. Humane endpoints were rigorously observed throughout the study. From Days 3–24 post-implantation, treatments were administered via intraperitoneal injection or oral gavage, including BCc1 at 0.1 or 0.4 mg/kg IP and 10 or 40 mg/kg orally, cyclophosphamide at 20 mg/kg IP, or PBS vehicle controls (detailed in Fig. 2). At study termination, animals were euthanized by CO_2_ inhalation followed by cervical dislocation under deep anesthesia in strict adherence to AVMA, CCAC, and EU Directive 2010/63/EU guidelines. Tumors were subsequently harvested aseptically for downstream analyses.

The sample size of ten mice per group was determined following a rigorous statistical review by an independent consultant. Based on prior in vivo tumor challenge and transplantation experiments from our group, eight animals per group were deemed sufficient to achieve adequate statistical power. To enhance the robustness of the analysis, the cohort size was increased to ten. This decision was supported by an a priori power analysis performed using G\∗Power (α = 0.05, power = 0.8, effect size = X) and validated through the resource equation method (E = N – s), which yielded an E-value within the accepted range of 10–20 for the current design involving seventy animals across seven groups. This approach aligns with established ethical and statistical.

All procedures were conducted under veterinary supervision and complied with institutional Animal Care and Use Committee approvals and international animal welfare standards.

### 2.15 RNA extraction and quality control

High-purity RNA was extracted from tumor samples using the YTzol Pure RNA Kit (YTA, Iran) according to the manufacturer's protocol. RNA concentration was determined via NanoDrop 2000 spectrophotometer (Thermo Scientific, Wilmington, USA) by measuring the A260/A280 ratio. To normalize RNA concentrations to 1000 ng, we applied the formula M1V1=M2V2. RNA quality was further assessed using agarose gel electrophoresis.

#### 2.15.1 Primer design and gene targets

Forward and reverse primers were designed for the following target genes: *Beclin-1*, *ATG-4B*, *ATG-7*, and *mTOR*, with *β-actin* as the internal control.

Primer sequences were carefully selected to ensure target specificity. The sequences for real-time PCR amplification were as follows:

*Beclin-1*: Forward: 5′-TCTCGCAGATTCCATCCCC-3'; Reverse: 5′-TCTTCGGCTGAGGTTCCTCAT-3'; Amplicon size: 111 bp
*ATG-4B*: Forward: 5′-GTGTCGCCATTCCATCCGCAC-3'; Reverse: 5′-AGGCTTCTGCTGAGCGACTTC-3'; Amplicon size: 190 bp
*ATG-7*: Forward: 5′-AAGGCGTCTCTCTCCAGTGCC-3'; Reverse: 5′-TTCAGCAGAGCCTGCCTCATG-3'; Amplicon size: 153 bp
*mTOR*:Forward: 5′-GCCAGCATCTAGCAACCGGAG-3'; Reverse: 5′-ATGTTCCAGCTGCTCATAGAAC-3'; Amplicon size: 168 bp
*β-actin (Internal Control)*: Forward: 5′-CTGCACCTGTGTCTCTGCCT-3'; Reverse: 5′-ATGTGAGCGACGATTCCC-3'; Amplicon size: 218 bp

#### 2.15.2 Real-time PCR

Real-time PCR amplification was performed using these primers to assess the specificity and efficiency of gene amplification.

### 2.16 cDNA synthesis

First-strand complementary DNA (cDNA) was synthesized from extracted RNA using RevertAid reverse transcriptase (Fermentas, Germany), following the manufacturer's instructions. Each 20 μL reaction contained 10 μL of RNA template, 1 μL each of dNTPs, random hexamer primers, and reverse transcriptase enzyme, 4 μL of 5 × reaction buffer, and 3 μL of nuclease-free water.

Thermal conditions included primer annealing at 25 °C for 3 min, reverse transcription at 42 °C for 60 min, and enzyme inactivation at 70 °C for 5 min.

### 2.17 Quantitative real-time PCR (qPCR)

Quantitative real-time PCR was used to analyze mRNA expression levels of *Beclin-1*, *ATG-4B*, *ATG-7*, mTOR, and the housekeeping gene *β-actin* in tumor tissue samples. cDNA was synthesized from extracted RNA and used as a template for amplification.

### 2.18 Reaction setup

Each 20 μL reaction contained 1 μL of cDNA, 12.5 μL of Real Q Plus 2 × Master Mix SYBR Green (Ampliqon), 1 μL of gene-specific primer mix, and 5.5 μL of nuclease-free water. Reactions were run in a rotary-based real-time PCR system.

### 2.19 Thermal cycling conditions

The qPCR protocol consisted of an initial denaturation at 95 °C for 15 min, followed by 40 cycles of 95 °C for 10 s, gene-specific annealing (*Beclin-1*: 59.5 °C; *ATG-4B*: 63.5 °C; *ATG-7*: 64 °C; *mTOR*: 60 °C; *β-actin*: 62 °C) for 30 s, and extension at 72 °C for 30 s. Melt curve analysis was performed post-amplification to confirm product specificity and detect minor sequence variations.

### 2.20 Statistical analysis

A priori sample size estimation was conducted using G\∗Power software (version 3.1.9.7) to inform the design of a one-way ANOVA. Based on a moderate-to-large effect size (Cohen's f = 0.4), α = 0.05, and a desired power of 0.80, the minimum required sample size was calculated to be eight animals per group. To account for inter-subject variability and enhance statistical robustness in gene expression and tumor analyses, ten animals per group were included, yielding a total of seventy mice across seven experimental groups. This sample size aligns with both ethical considerations and statistical guidelines, including the resource equation method (E = N – s), which confirmed sufficient experimental power.

Statistical analyses in this research were performed using GraphPad Prism software, version 6.01. Data interpretation involved one-way analysis of variance (ANOVA), followed by Tukey's HSD post hoc test to identify significant differences in gene expression between control and treated groups. Statistical significance was established at P-values below 0.05. Results were expressed as mean ± standard deviation (SD) and processed using Excel software. Real-time PCR analysis relied on threshold cycle comparisons, with differences in threshold cycles calculated between treated (drug-exposed) and control (untreated) groups. The relative expression levels of target genes, normalized to the reference gene *β-actin*, were quantified using the ΔΔCT method and the $2^{-\Delta \Delta CT}$ formula.

## 3 Results

### 3.1 Dynamic light scattering results

**Fig. 1:**
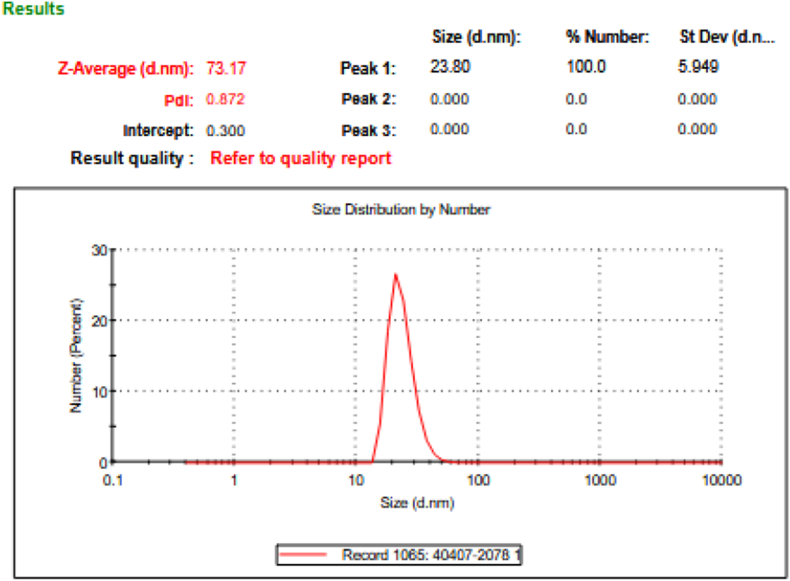
Dynamic light scattering profile of BCc1 nanomedicine. Representative DLS analysis demonstrating the hydrodynamic size distribution of BCc1 nanoparticles dispersed in aqueous medium. The primary particle population exhibits a mean diameter of 73.17 nm, as identified by the dominant peak in the number-weighted distribution. The formulation displays a polydispersity index (PDI) of 0.872, reflecting a broad yet analytically interpretable nanoparticle distribution profile. Measurements were performed at 25 °C using water as the dispersant, with a recorded count rate of 1065 kcps, ensuring adequate scattering intensity for reliable detection. These results confirm the nanoscale integrity and colloidal stability of the BCc1 formulation.

Dynamic light scattering measurements revealed that BCc1 nanoparticles formed a stable colloidal dispersion in aqueous medium, exhibiting a hydrodynamic diameter indicative of nanoscale integrity. The particle population demonstrated a mean diameter of 73.17 nm, which is consistent with the nanoscale dimensions previously reported for related nano-chelated iron structures. The formulation displayed a polydispersity index (PDI) of 0.872, reflecting a broad yet analyzable distribution profile characteristic of complex nanostructured systems. Analysis of the scattering intensity curves further indicated that the dominant particle population accounted for approximately 58.6 % of the total signal, suggesting the presence of a major structural fraction within the dispersion (Fig. 1). These observations collectively support the physicochemical stability of BCc1 in aqueous suspension and align with the general size range reported by Fakharzadeh et al. for similar nanochelated frameworks.

**Fig. 2:**
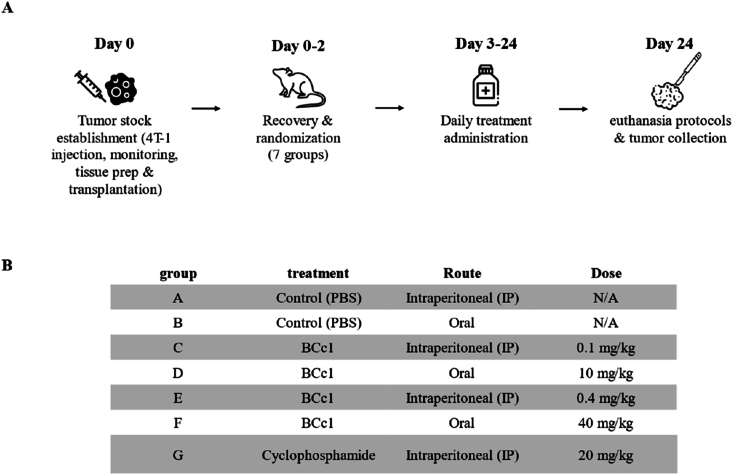
Experimental timeline and treatment groups. Seventy female BALB/c mice were randomized into seven treatment groups (n = 10 per group) following a comprehensive sample size determination strategy. (A) Schematic representation of the experimental timeline, outlining the key procedural steps: tumor stock establishment (Day 0), recovery and randomization of animals into seven treatment groups (Days 0–2), daily treatment administration (Days 3–24), and euthanasia protocols followed by tumor collection (Day 24). (B) Treatment groups and corresponding administration details. Mice were assigned to seven groups, with treatments including PBS (control), BCc1 at varying doses (0.1, 0.4, 10, and 40 mg/kg) administered either intraperitoneally (IP) or orally, and cyclophosphamide (20 mg/kg, IP). Group A and B received PBS via IP or oral routes, respectively.

### 3.2 Physicochemical justification for BCc1 systemic administration

Previous physicochemical evaluations have demonstrated that BCc1 exhibits excellent aqueous solubility and exceptional acid stability [19,23], supporting the feasibility of systemic administration.

### 3.3 *Beclin-1* gene expression in tumor microenvironment (TME)

The expression levels of *Beclin-1* were significantly elevated in certain treatment groups within the tumor microenvironment (TME). Specifically, the cyclophosphamide injection group (1.691 ± 0.2570) exhibited a notable increase in *Beclin-1* expression compared to the PBS injection control group (1.000 ± 0; P = 0.0218). Similarly, the 10 mg/kg oral BCc1 group showed a significant upregulation of *Beclin-1* expression (1.692 ± 0.2128) relative to the oral PBS control group (1.000 ± 0; P = 0.0216), indicating a dose-dependent response to BCc1 treatment in the TME.

In contrast, the 0.1 mg/kg BCc1 injection group demonstrated a non-significant increase in *Beclin-1* expression (1.285 ± 0.1907) when compared to the PBS-injected control group (1.000 ± 0; P = 0.6861), suggesting that the lowest dose of BCc1 did not significantly affect *Beclin-1* expression. The 0.4 mg/kg BCc1 injection group also showed a non-significant elevation in *Beclin-1* gene expression (1.599 ± 0.1257) relative to the PBS control (1.000 ± 0; P = 0.0549), while the 40 mg/kg oral BCc1 group (1.600 ± 0.4126) displayed a non-significant increase compared to the oral PBS control (0.000 ± 0; P = 0.0543), suggesting that higher doses of BCc1 may induce a modest upregulation of *Beclin-1* expression in the TME.

**Fig. 3:**
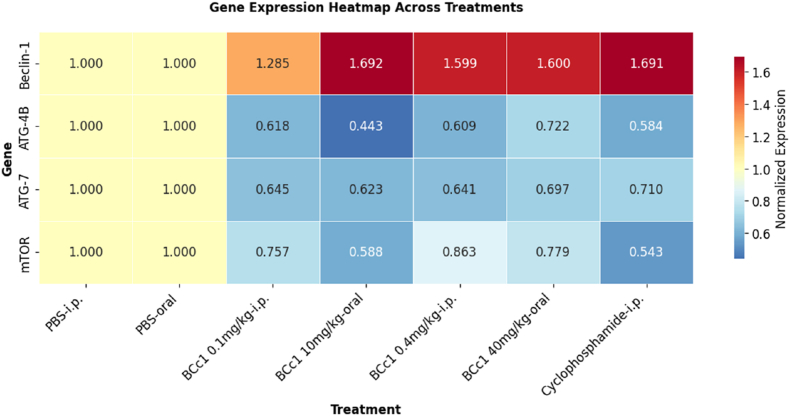
Heatmap of normalized gene expression across experimental treatments. Heatmap illustrating the normalized expression levels of *Beclin-1*, *ATG-4B*, *ATG-7*, and *mTOR* genes under various treatment conditions, including PBS (intraperitoneal and oral), BCc1 at different doses (0.1, 0.4 mg/kg i.p.; 10, 40 mg/kg oral), and cyclophosphamide (i.p.). Expression values were normalized to the PBS-i.p. control group. The color scale represents relative gene expression, where warmer colors indicate upregulation and cooler colors indicate downregulation compared to control.

Comparative analysis between the 0.1 mg/kg BCc1 injection group and the 0.4 mg/kg BCc1 injection group revealed no significant difference in *Beclin-1* gene expression (1.285 ± 0.1907 vs. 1.599 ± 0.1257; P = 0.5890), suggesting a similar effect on *Beclin-1* expression across these two doses. Similarly, the 0.1 mg/kg BCc1 injection group showed a non-significant reduction in *Beclin-1* expression when compared to the cyclophosphamide injection group (1.285 ± 0.1907 vs. 1.691 ± 0.2570; P = 0.3177). For oral BCc1 treatments, the 10 mg/kg BCc1 group (1.692 ± 0.2128) exhibited no significant difference in *Beclin-1* expression relative to the 40 mg/kg BCc1 group (1.600 ± 0.4126; P = 0.9982), indicating that the upregulation of *Beclin-1* is not dose-dependent at these oral doses. Additionally, the 0.4 mg/kg BCc1 injection group (1.599 ± 0.1257) displayed a non-significant reduction in *Beclin-1* expression compared to the cyclophosphamide injection group (1.691 ± 0.2570; P = 0.9982) (Fig. 3).

These results were quantified using quantitative real-time PCR, with data normalized to *β-actin* and expressed as mean ± SD from n = 3 biological replicates per group. Statistical comparisons were performed using one-way ANOVA followed by Tukey's post hoc test, with significance defined as P < 0.05, P < 0.01, and P < 0.001. A significant increase in *Beclin-1* expression was observed in the 10 mg/kg oral BCc1 group and the cyclophosphamide i.p. group compared to their respective controls, as indicated by P < 0.05, and P < 0.01, respectively.

### 3.4 *ATG-4B* gene expression in TME

The expression levels of *ATG-4B*, a key autophagy-related gene, were significantly reduced across several treatment groups when compared to their respective control groups. Specifically, the 0.1 mg/kg BCc1 injection group (0.6182 ± 0.05362), 0.4 mg/kg BCc1 injection group (0.6090 ± 0.06404), and cyclophosphamide injection group (0.5835 ± 0.1263) exhibited significant decreases in *ATG-4B* gene expression relative to the PBS injection control group (1.000 ± 0; P = 0.0003, P = 0.0002, and P = 0.0001, respectively), suggesting a strong suppression of *ATG-4B* in response to these treatments. In addition, the oral BCc1 treatment groups at 10 mg/kg (0.04832 ± 0.4425) and 40 mg/kg (0.1102 ± 0.7223) also showed significant downregulation of *ATG-4B* expression when compared to the oral PBS control group (0.000 ± 0; P < 0.0001 and P = 0.0053, respectively). These findings indicate that both BCc1 and cyclophosphamide treatments, regardless of administration route, effectively inhibit *ATG-4B* gene expression in the TME, further supporting their role in modulating autophagy processes.

A comparative analysis of the 0.1 mg/kg BCc1 injection group relative to the 0.4 mg/kg BCc1 injection group revealed a non-significant increase in *ATG-4B* expression (0.6182 ± 0.05362 vs. 0.6090 ± 0.06404; P > 0.9999), suggesting that the lower dose of BCc1 does not notably differ from the higher dose in influencing *ATG-4B* expression. Similarly, there was no significant difference in *ATG-4B* expression between the 0.1 mg/kg BCc1 injection group and the cyclophosphamide injection group (0.6182 ± 0.05362 vs. 0.5835 ± 0.1263; P = 0.9964), nor between the 0.4 mg/kg BCc1 injection group and the cyclophosphamide injection group (0.6090 ± 0.06404 vs. 0.5835 ± 0.1263; P = 0.9994), suggesting that the effects of BCc1 at both doses are comparable to cyclophosphamide in terms of suppressing *ATG-4B* expression in the TME (Fig. 3).

These results were obtained by quantitative real-time PCR, with data normalized to *β-actin* and expressed as mean ± SD from n = 3 biological replicates per group. Statistical significance was assessed using one-way ANOVA followed by Tukey's post hoc test, with P values of P < 0.05, P < 0.01, and P < 0.001 indicating statistically significant differences between treatment and control groups. The findings demonstrate a robust downregulation of *ATG-4B* expression across various treatment conditions, with BCc1 and cyclophosphamide showing particularly strong effects, further implicating autophagy-related genes as potential therapeutic targets in the tumor microenvironment.

### 3.5 *ATG-7* gene expression in TME

The expression levels of *ATG-7*, an essential gene involved in autophagy regulation, were significantly reduced across multiple treatment groups compared to their respective controls. Specifically, both the 0.1 mg/kg BCc1 injection group (0.6450 ± 0.1578) and the 0.4 mg/kg BCc1 injection group (0.6407 ± 0.05973) showed significant decreases in *ATG-7* expression relative to the PBS injection control group (1.000 ± 0; P = 0.0010 and P = 0.0009, respectively). Similarly, oral BCc1 treatments at 10 mg/kg (0.06239 ± 0.6230) and 40 mg/kg (0.6965 ± 0.08950) significantly reduced *ATG-7* gene expression compared to the oral PBS control group (0.000 ± 0; P = 0.0006 and P = 0.0043, respectively). The cyclophosphamide injection group also demonstrated a significant reduction in *ATG-7* expression (0.04547 ± 0.7101) compared to the PBS injection group (0.000 ± 0; P = 0.0063). These findings suggest that both BCc1 and cyclophosphamide treatments effectively suppress *ATG-7* expression within the tumor microenvironment, potentially altering autophagy-mediated processes in cancer cells.

However, when comparing 0.1 mg/kg BCc1 and 0.4 mg/kg BCc1 injections, no significant difference in *ATG-7* expression was observed (0.6450 ± 0.1578 vs. 0.6407 ± 0.05973; P > 0.9999), suggesting that both doses exert a similar level of suppression. Similarly, no significant difference was found between the 0.1 mg/kg BCc1 injection group and the cyclophosphamide injection group (0.6450 ± 0.1578 vs. 0.04547 ± 0.7101; P = 0.9395), further supporting the comparable effects of BCc1 and cyclophosphamide in reducing *ATG-7* expression. In the oral BCc1 groups, no significant difference was observed between the 10 mg/kg BCc1 group (0.6230 ± 0.06239) and the 40 mg/kg BCc1 group (0.6965 ± 0.08950; P = 0.8992), indicating a lack of dose-dependent effect on *ATG-7* expression. Finally, the 0.4 mg/kg BCc1 injection group showed no significant difference compared to the cyclophosphamide injection group (0.6407 ± 0.05973 vs. 0.04547 ± 0.7101; P = 0.9203) (Fig. 3).

Quantitative real-time PCR was employed to measure *ATG-7* gene expression, with data normalized to *β-actin*. The results are presented as mean ± SD from n = 3 biological replicates per group, with error bars representing standard deviation. Statistical analysis was performed using one-way ANOVA, followed by Tukey's post hoc test, with significance defined as P < 0.05, P < 0.01, and P < 0.001. All treatment groups demonstrated a significant reduction in *ATG-7* expression compared to the control groups (PBS-oral and PBS-i.p.), confirming the inhibitory effects of BCc1 and cyclophosphamide on autophagy-related pathways within the TME.

### 3.6 *mTOR* gene expression in TME

The expression levels of the *mTOR* gene were significantly downregulated in several treatment groups compared to the PBS injection group. Specifically, the 0.1 mg/kg BCc1 injection group (0.7570 ± 0.02529), cyclophosphamide injection group (0.5432 ± 0.01560), 10 mg/kg oral BCc1 group (0.5875 ± 0.07552), and 40 mg/kg oral BCc1 group (0.7794 ± 0.01830) all exhibited significant reductions in *mTOR* expression compared to the PBS injection group (1.000 ± 0; P < 0.0001). These results indicate that BCc1 and cyclophosphamide treatments, regardless of administration route, effectively suppress *mTOR* expression in the tumor microenvironment, suggesting potential effects on *mTOR*-driven signaling pathways in tumor progression.

Additionally, the 0.4 mg/kg BCc1 injection group showed a notable decrease in *mTOR* gene expression (0.8630 ± 0.04119) compared to the PBS injection group (0.000 ± 0; P = 0.0042), further supporting the downregulatory impact of BCc1 on *mTOR* signaling.

When comparing the cyclophosphamide injection group to the BCc1-treated groups, the cyclophosphamide group exhibited a more pronounced suppression of *mTOR* expression, with a significant decrease compared to the 0.1 mg/kg BCc1 injection group, 0.4 mg/kg BCc1 injection group, and 40 mg/kg oral BCc1 group (0.5432 ± 0.01560 vs. 0.7570 ± 0.02529, 0.8630 ± 0.04119, and 0.7794 ± 0.01830; P < 0.0001), indicating that cyclophosphamide may have a stronger inhibitory effect on *mTOR* in the TME (Fig. 3).

Quantitative real-time PCR was used to assess *mTOR* gene expression, with data normalized to *β-actin* and presented as mean ± SD from n = 3 biological replicates per group. Statistical significance was assessed by one-way ANOVA, followed by Tukey's post hoc test, with significance defined as P < 0.05, P < 0.01, and P < 0.001. All Treatment groups exhibited variable downregulation of *mTOR* expression, with cyclophosphamide and 40 mg/kg oral BCc1 showing the most pronounced decrease relative to the combined control groups (PBS-oral and PBS-i.p.).

## 4 Discussion

Breast cancer (BC) continues to pose a formidable clinical challenge, demanding innovative therapeutic strategies beyond conventional modalities, which often demonstrate limited efficacy. Recent advancements in immunotherapy have underscored the potential for selective anti-tumor responses while minimizing off-target toxicity [24]. Within this landscape, nanoparticle-based systems have emerged as versatile drug delivery platforms, offering enhanced bioactivity, favorable pharmacokinetics, and the ability to interact dynamically with the TME [25].

BCc1 nanoparticles, engineered using advanced nanochelating technology, constitute a novel therapeutic approach. By modulating ion homeostasis, particularly iron metabolism, BCc1 not only induces direct cytotoxicity but also engages innate immune signaling pathways to potentiate anti-tumor responses [26,27]. This chelating activity enables BCc1 to influence the TME, altering key cellular processes implicated in tumor progression. Preclinical studies have demonstrated BCc1's efficacy in tumor suppression, with unique biodistribution properties allowing the selective sequestration and excretion of excess metal ions via hepatic and urinary systems [19].

Early clinical translation in gastric cancer patients, including both metastatic and non-metastatic cohorts, has revealed improved survival and reduced tumor burden, highlighting BCc1's translational promise [16,17].

The hydrodynamic size and distribution profile of BCc1 obtained in this study, measured using a laser diffraction–based detection system with multiple-scattering capability under controlled ambient conditions, align closely with the physicochemical characteristics previously reported for structurally related nano-chelated iron complexes. Comparative evaluation of our DLS parameters with reference datasets reported in studies [23,28] indicates a similar colloidal behavior and size distribution pattern, thereby supporting the expected aqueous stability and dispersion profile of BCc1.

Autophagy and apoptosis are intricately connected cellular processes whose roles in cancer are highly context-dependent. Autophagy can act cytoprotectively, supporting tumor survival under stress, or promote cell death when excessively activated [[29], [30], [31]].

Crosstalk with apoptotic pathways, mediated through shared signaling networks, further complicates the functional outcome [32]. In cancer, autophagy may facilitate tumor growth by supplying metabolic intermediates [33], while simultaneously exerting tumor-suppressive effects via removal of damaged organelles and maintenance of genomic integrity [34]. The dualistic role of autophagy varies by cancer type: it is generally tumor-suppressive in prostate and BC [35] but can drive progression in pancreatic and colorectal cancers, highlighting opportunities for therapeutic modulation [36].

Our study sought to delineate the molecular underpinnings of BCc1's anti-tumor effects in a BALB/c murine model of BC, focusing on key autophagy regulators (*Beclin-1*, *ATG-4B*, *ATG-7*) and the *mTOR* signaling axis. Tumor growth analyses confirmed significant suppression in BCc1-treated groups, particularly at a 10 mg/kg oral dose, consistent with prior observations in murine models [15,22].

*Beclin-1*, a central initiator of autophagy, exhibited context-dependent modulation within the breast TME. While downregulation in malignant epithelial cells compromises autophagy-mediated genomic stability, stromal mesenchymal cells may upregulate *Beclin-1*, promoting metabolic recycling, extracellular matrix remodeling, and immune modulation changes often correlated with adverse outcomes [[37], [38], [39]]. BCc1 treatment significantly increased *Beclin-1* expression, comparable to cyclophosphamide-induced modulation, suggesting a nuanced mechanism that balances autophagy activation for tumor suppression without triggering cytoprotective resistance. These findings advance prior work by demonstrating that BCc1's regulation of *Beclin-1* is both dose- and route-dependent, offering a refined understanding of its therapeutic window [16,22].

Quantitative PCR (qPCR) analysis revealed that the TME exerts a regulatory influence on *Beclin-1* gene expression. Cyclophosphamide treatment significantly upregulated *Beclin-*1 mRNA levels relative to controls, indicating a potential role in enhancing *Beclin-1* expression. Similarly, oral BCc1 administration at 10 mg/kg significantly increased *Beclin-1* expression compared with untreated controls. Lower doses of parenteral BCc1 (0.1 mg/kg and 0.4 mg/kg) elicited non-significant increases in *Beclin-1* expression, suggestive of a possible dose-dependent trend. However, no significant differences were observed between the two parenteral BCc1 dose groups, indicating that factors other than dosage may modulate these effects.

When comparing administration routes, oral BCc1 at 10 mg/kg produced a non-significant increase in *Beclin-1* expression relative to the 40 mg/kg oral dose, suggesting that both dosage and route of delivery influence *Beclin-1* regulation. Furthermore, the 0.4 mg/kg parenteral BCc1 group exhibited a non-significant decrease in *Beclin-1* expression compared with the cyclophosphamide group, indicating potential mechanistic differences between these interventions. Consistent with prior reports, our qPCR data confirm that BCc1 treatment upregulates *Beclin-1* expression. These findings support the hypothesis that BCc1 nanomedicine may exert tumor-suppressive effects, at least in part, by activating autophagy within the TME [16,22].

*ATG-4B*, a cysteine protease critical for autophagy, exhibits complex and context-dependent roles in oncogenesis, functioning either as a tumor suppressor or as a facilitator of tumor progression depending on cellular and microenvironmental conditions [40,41]. In BC, *ATG-4B* represents the predominant isoform, and its pro-survival activity renders it a particularly attractive therapeutic target [42].

To elucidate the regulatory influence of BCc1 nanomedicine on *ATG-4B* expression, quantitative PCR analyses were performed using normalized control groups. Both parenteral BCc1 at 0.1 mg/kg and 0.4 mg/kg, as well as cyclophosphamide, produced significant downregulation of *ATG-4B* mRNA relative to PBS-treated controls. A comparable suppressive pattern was observed following oral BCc1 administration. No statistically significant differences were detected between the two parenteral BCc1 doses, nor between either parenteral BCc1 group and cyclophosphamide treatment, indicating a consistent effect across administration routes and doses [[41], [42], [43]].

These results indicate that BCc1, regardless of administration route, significantly reduces *ATG-4B* expression in breast tumor cells, with a magnitude of suppression comparable to cyclophosphamide—a chemotherapeutic known to activate autophagy as part of the cellular stress response. This downregulation suggests that BCc1 may impair autophagy-dependent survival mechanisms exploited by malignant cells. Notably, unlike *Beclin-1*, which is contextually activated to facilitate tumor-suppressive autophagy, *ATG-4B* suppression by BCc1 represents a selective inhibition of pro-survival autophagic flux. This dualistic regulation likely reflects a nuanced mechanistic modulation, suggesting that BCc1 may fine-tune autophagic pathways to favor anti-tumor outcomes—a property not previously reported in nanochelating therapeutics [15,41]. Therapeutically, the selective suppression of *ATG-4B* by BCc1 seems to carry multiple implications. First, it may contribute to its anti-neoplastic efficacy by attenuating autophagy-mediated tumor cell survival [15]. Second, the apparent parallel modulation of *ATG-4B* by both BCc1 and cyclophosphamide suggests a potential pharmacodynamic convergence that could be strategically leveraged in combination regimens to achieve synergistic targeting of autophagy-dependent tumor resilience. Collectively, these findings suggest that BCc1 may act as a dual-function nanomedicine, capable of modulating tumor-suppressive autophagy (via *Beclin-1*) while inhibiting pro-survival autophagy (via *ATG-4B*). Further protein and functional validation is required to conclusively confirm these mechanisms and will be a focus of future research.

*ATG-7*, a pivotal E1-like enzyme in the autophagic cascade, is frequently overexpressed within the breast TME and plays a central role in autophagosome formation, tumor growth, invasion, and immune evasion [[43], [44], [45]]. While *ATG-7* is often implicated in tumor initiation and progression, its functional profile is highly context dependent, reflecting the dualistic nature of autophagy as both a tumor-suppressive and tumor-promoting process [46,47]. Consequently, a comprehensive understanding of *ATG-7* regulation across distinct BC subtypes is critical for the rational design of autophagy-targeted therapies.

To investigate the impact of BCc1 nanomedicine on *ATG-7* expression, quantitative PCR analyses were performed in tumor-bearing BALB/c mice. Parenteral BCc1 at 0.1 mg/kg and 0.4 mg/kg, as well as oral BCc1 at 10 mg/kg and 40 mg/kg, elicited significant downregulation of *ATG-*7 mRNA relative to PBS-treated controls. Cyclophosphamide, a standard chemotherapeutic known to induce autophagy-associated stress responses, produced a comparable magnitude of suppression [[44], [45], [46], [47]]. No statistically significant differences were observed between the two parenteral BCc1 doses, nor between either BCc1 dose and cyclophosphamide. Similarly, *ATG-7* expression did not differ significantly between the two oral BCc1 doses or between the 0.4 mg/kg parenteral BCc1 and cyclophosphamide groups.

These findings indicate that BCc1 consistently suppresses *ATG-7* expression irrespective of administration route, highlighting its capacity to attenuate a critical survival pathway exploited by tumor cells. Notably, this selective inhibition occurs alongside the context-dependent upregulation of *Beclin-1*, suggesting that BCc1 can concurrently promote tumor-suppressive autophagy while inhibiting pro-survival autophagic flux. This dual regulatory effect distinguishes BCc1 from conventional chemotherapeutics and positions it as a refined, context-specific modulator of autophagy in BC [15,[44], [45], [46], [47]].

Therapeutically, the downregulation of *ATG-*7 by BCc1 holds several implications. First, it may directly impair tumor cell survival by disrupting adaptive autophagy under metabolic and therapeutic stress. Second, the convergence of BCc1 and cyclophosphamide effects suggests potential opportunities for combination regimens, enabling dual interference with autophagy-dependent tumor resilience. Collectively, these results reinforce the role of BCc1 as a potent nanomedicine capable of modulating autophagic pathways to achieve anti-tumor efficacy, in alignment with prior observations [15].

Quantitative PCR (qPCR) analysis was performed to elucidate the regulatory effects of BCc1 nanomedicine on *mTOR* gene expression within the breast TME. The mechanistic target of rapamycin (*mTOR*) is a central regulator of cellular growth, metabolism, and survival, frequently hyperactivated in BC, where it drives tumorigenesis, promotes angiogenesis, and facilitates immune evasion [48]. Dysregulated *mTOR* signaling is intricately embedded within the phosphatidylinositol 3-kinase (PI3K)/protein kinase B (Akt) pathway and reinforced through multiple feedback loops, collectively contributing to therapeutic resistance [49].

Although pharmacological *mTOR* inhibition has demonstrated therapeutic promise, durable clinical benefit is often limited by the complexity of the TME and adaptive resistance mechanisms, highlighting the need for integrative strategies, such as combining *mTOR* inhibitors with immune checkpoint blockade, to enhance antitumor efficacy [50].

In this study, BCc1 nanomedicine significantly modulated *mTOR* transcript levels across multiple treatment cohorts. Parenteral BCc1 at 0.1 mg/kg and 0.4 mg/kg produced marked suppression of *mTOR* expression relative to PBS controls, while oral BCc1 at 10 mg/kg and 40 mg/kg elicited substantial decreases in *mTOR* mRNA compared with the oral PBS group. Cyclophosphamide treatment induced a more pronounced reduction in *mTOR* levels compared with all BCc1-treated groups. No statistically significant differences were observed between the two parenteral BCc1 doses, whereas cyclophosphamide remained significantly more suppressive. These results indicate a consistent downregulation of *mTOR* signaling following BCc1 exposure, albeit to a lesser extent than cyclophosphamide, suggesting that BCc1 may act through alternative upstream regulators or parallel signaling pathways [[48], [49], [50]].

Functionally, BCc1-mediated *mTOR* inhibition carries significant implications for tumor suppression. By attenuating *mTOR* activity, BCc1 may impede tumor growth, restrict angiogenic signaling, and enhance tumor responsiveness to immunotherapeutic interventions, including checkpoint blockade. Importantly, these effects complement BCc1's autophagy-modulatory actions, positioning the nanomedicine as a dual-function anticancer agent capable of simultaneously targeting metabolic and autophagic tumor survival mechanisms [15,[48], [49], [50]].

The differential magnitude of *mTOR* suppression between BCc1 and cyclophosphamide also provides a mechanistic rationale for combination regimens, potentially enabling synergistic inhibition of tumor growth while mitigating adaptive resistance. Collectively, these findings establish BCc1 nanomedicine as a promising therapeutic candidate, with transcriptional evidence supporting its immunomodulatory and autophagy-targeted mechanisms of action. Nevertheless, protein-level analyses of autophagy, metabolic contributions, and translational safety profiles remain to be investigated, warranting integrated proteomic, metabolic, and toxicological studies to fully elucidate BCc1's therapeutic potential across diverse malignancies.

The combined physicochemical evidence indicates that BCc1 possesses a highly robust and pharmacologically favorable profile that directly supports the rationale for its selected administration route. Across all evaluated conditions—including physiologic pH and strongly acidic environments—BCc1 maintained its structural integrity with remarkable consistency. Spectroscopic assessments demonstrated that the nanodrug preserved approximately 99.25 % of its native characteristics following 1 h of exposure at 37 °C, with no detectable signatures of acid-induced degradation. This stability, achieved without any auxiliary stabilizing excipients, underscores the intrinsic strength of its ionic nanostructure and its capacity to endure the harsh acidic milieu typically encountered during gastrointestinal transit [23].

Equally important, BCc1 exhibited complete aqueous solubility from pH 2.0 to 7.4, remaining fully dispersed without precipitation, sedimentation, or observable aggregation. Such a uniformly soluble profile minimizes formulation-related challenges and ensures predictable dispersion throughout both gastric and systemic compartments [19,23].

Taken together, the exceptional acid resistance and broad-range solubility of BCc1 suggest that the nanodrug can retain its physicochemical integrity throughout gastrointestinal passage, support efficient systemic uptake, and transition reliably into downstream metabolic processes, primarily hepatic. These characteristics collectively point to the potential for consistent bioavailability and dependable therapeutic performance, reinforcing BCc1's suitability for continued preclinical investigation and advancement toward translational cancer nanomedicine applications.

A more definitive mechanistic interpretation of BCc1's biological activity will require complementary protein-level validation. While the present work emphasizes transcriptional modulation, rigorous confirmation of Beclin-1, LC3-I/II, p62/SQSTM1, *ATG4B*, *ATG7*, and phosphorylated and total *mTOR* through full-length immunoblotting—with appropriate molecular-weight markers, loading controls, and quantitative replicates—is essential for distinguishing whether BCc1 influences autophagy initiation, maturation, or both. Likewise, direct assessment of autophagic flux using agents such as bafilomycin A1 or chloroquine, together with ultrastructural visualization of autophagosomes and autolysosomes by transmission electron microscopy, would provide critical discriminatory power to differentiate enhanced autophagic activity from impaired turnover. As these experiments were not conducted within the scope of the current study, we explicitly acknowledge this as a methodological limitation.

Even with this constraint, comparative analysis with the existing literature highlights the distinctive nature of BCc1's mechanistic profile. In contrast to most nanoparticle-based therapeutics, which predominantly rely on cytotoxic payloads or broad immunomodulatory effects [4,6,25,51], BCc1's nanochelation framework appears to engage a more sophisticated regulatory axis—potentially enhancing tumor-suppressive Beclin-1 signaling while concurrently attenuating pro-survival *ATG4B* and *ATG7*, alongside downregulation of the *mTOR* pathway. This integrated pattern of autophagy modulation may represent a meaningful advancement beyond the mechanistic capacity of conventional chemotherapeutics and previously developed nanodrugs. The emerging evidence suggests that BCc1 may exert a coordinated influence on immune function and tissue-specific autophagy, thereby strengthening its candidacy as a multifunctional anticancer agent.

To fully delineate this mechanistic landscape, future investigations incorporating comprehensive proteomic, metabolic, and toxicological approaches—together with the aforementioned flux and protein-expression assays—will be essential. Such studies will be critical to refine the mechanistic model proposed here and to support the translational progression of BCc1 across a spectrum of malignancies.

## 5 Conclusion

This study provides the first in vivo evidence that nanochelate-based BCc1 modulates autophagy-associated genes (*Beclin-1*, *ATG-4B*, *ATG-7*) and the *mTOR* signaling pathway in a BALB/c BC model. BCc1 exhibits a dual regulatory effect—upregulating tumor-suppressive *Beclin-1* while downregulating pro-survival *ATG-4B*, *ATG-7*, and *mTOR*—in a manner dependent on dose and administration route. This pathway-selective modulation likely underpins its observed anti-tumor efficacy and represents a novel mechanism of action not previously reported for BC nanotherapeutics.

The comparable effects of BCc1 and cyclophosphamide—a standard chemotherapeutic—underscore BCc1's translational potential and support its rational integration into combination therapy regimens. By simultaneously promoting context-specific autophagy, suppressing key survival pathways, and exerting immunomodulatory effects, BCc1 offers multiple therapeutic advantages, including the potential to overcome drug resistance, minimize systemic toxicity, and enhance the efficacy of existing treatments.

These findings provide a robust mechanistic foundation for further preclinical and clinical studies. Future investigations should focus on integrative proteomic, metabolic, and toxicological analyses to fully characterize BCc1's molecular mechanisms, optimize dosing and administration strategies, and evaluate long-term safety and efficacy across diverse BC subtypes. Collectively, this integrative profile positions BCc1 as a unique and promising nanomedicine platform for targeted, pathway-specific interventions in BC therapy.

## Data availability statement

All data used for the analyses in this report are available from the corresponding author on reasonable request.

## Ethics approval and consent to participate

All experimental procedures involving animals were conducted in strict accordance with institutional and national ethical standards and received formal approval from the Institutional Animal Ethics Committee of the Islamic Azad University of Tehran Medical Sciences (Approval code: IR.IAU.PS.REC.1400.341).

## Funding

This work was partially supported by Cancer Research Center of 10.13039/501100005851Shahid Beheshti University of Medical Sciences, Tehran, Iran (Grant No. 28100, IR.SBMU.CRC.REC.1400.033).

## CRediT authorship contribution statement

**Fereshteh Moheb Afzali:** Investigation, Validation, Writing – original draft. **Masoumeh Heshmati:** Investigation. **Ali Salimi:** Investigation. **Somayeh Kalanaky:** Data curation, Resources, Validation. **Saideh Fakharzadeh:** Data curation, Resources. **Maryam Hafizi:** Data curation, Resources. **Mohammad Esmail Akbari:** Funding acquisition, Project administration, Supervision. **Mohammad Hassan Nazaran:** Methodology, Resources. **Mehrdad Hashemi:** Conceptualization, Investigation, Methodology, Supervision, Writing – original draft.

## Declaration of competing interest

The authors declare that they have no competing interests.

## Abbreviations

BC, Breast cancer; IP, intraperitoneal; NPs, Nanoparticles; ATG, autophagy-related; mTOR, mechanistic target of rapamycin; FBS, fetal bovine serum; SPF, specific pathogen-free; NOAEL, no-observed-adverse-effect level; cDNA, complementary DNA; qPCR, quantitative PCR; SD, mean ± standard deviation; TME, tumor microenvironment; PI3K/Akt, Phosphatidylinositol-3-kinase (PI3K)/protein kinase B (Akt); PDI, polydispersity index; DLS, dynamic light scattering.
