# Corrigendum to “Nanochelate-based BCc1 delivery and its impact on key regulatory pathways in BALB/c breast cancer: An analysis of Beclin-1, ATG-4B, ATG-7, and mTOR expression” [Biochem. Biophys. Rep. 45 (2026) 102418]

**DOI:** 10.1016/j.bbrep.2026.102751

**Published:** 2026-08-17

**Authors:** Fereshteh Moheb Afzali, Masoumeh Heshmati, Ali Salimi, Somayeh Kalanaky, Saideh Fakharzadeh, Maryam Hafizi, Mohammad Esmail Akbari, Mohammad Hassan Nazaran, Mehrdad Hashemi

**Affiliations:** a Department of Cellular and Molecular Biology, TeMS.C., Islamic Azad University, Tehran, Iran; b Cellular and Molecular Research Center, Iran University of Medical Sciences, Tehran, Iran; c Department of Research and Development, Sodour Ahrar Shargh Company, Tehran, Iran; d Cancer Research Center, Shahid Beheshti University of Medical Sciences, Tehran, Iran; e Farhikhtegan Medical Convergence Sciences Research Center, Farhikhtegan Hospital., Faculty of Medicine., TeMS.C., Islamic Azad University, Tehran, Iran; f Department of Genetics, Faculty of Medicine, TeMS.C., Islamic Azad University, Tehran, Iran

The authors regret that several errors were inadvertently introduced during the final preparation and proofreading of the published article.

First, several primer sequences and corresponding amplicon lengths reported in Section 2.15.1 ("Primer design and gene targets") were incorrectly transcribed. The quantitative real-time PCR experiments were performed using the correct primer sets, whereas incorrect primer sequences were inadvertently reported in the published manuscript. The corrected primer sequences and amplicon information are provided in the revised table accompanying this Corrigendum. These corrections do not affect the experimental procedures, raw data, ΔΔCt calculations, statistical analyses, data interpretation, or the conclusions of the study.

**Table 1:** Correct primer sequences and corresponding amplicon lengths replacing those incorrectly reported in the published article.

Target gene	Published primer sequences	Correct primer sequences	Amplicon length (bp)
***Beclin-1***	Forward: 5'-TCTCGCAGATTCCATCCCC-3'	Forward: 5'-ACCGTGTCACCATCCAGGAA-3'	185
	Reverse: 5'-TCTTCGGCTGAGGTTCCTCAT-3'	Reverse: 5'-GAAGCTGTTGGCACTTTCTGT-3'	
***ATG-4B***	Forward: 5'-GTGTCGCCATTCCATCCGCAC-3'	Forward: 5'-GTGTCACCGCATTCCATCCAC-3'	190
	Reverse: 5'-AGGCTTCTGCTGAGCGACTTC-3'	Reverse: 5'-AGGCCTTCTGATGAGCGACTTC-3'	
***ATG-7***	Forward: 5'-AAGGCGTCTCTCTCCAGTGCC-3'	Forward: 5'-AAGGTCGTGTCTGTCAAGTGCC-3'	153
	Reverse: 5'-TTCAGCAGAGCCTGCCTCATG-3'	Reverse: 5'-TTCATACAGAGGCTGCCTCACG-3'	
***mTOR***	Forward: 5'-GCCAGCATCTAGCAACCGGAG-3'	Forward: 5'-GCCACATCTAGCAACGTGAG-3'	168
	Reverse: 5'-ATGTTCCAGCTGCTCATAGAAC-3'	Reverse: 5'-ATGGTTCAGCTGGTCATAGAAG-3'	
***β-actin (Internal Control)***	Forward: 5'-CTGCACCTGTGTCTCTGCCT-3'	Forward: 5'-CGATATCGCTGCGCTGGTC-3'	219
	Reverse: 5'-ATGTGAGCGACGATTCCC-3'	Reverse: 5'-ACAATGCCATGTTCAATGGGG-3'	

Second, the animal ethics approval identifier was incorrectly reported. The published article cited approval code **IR.IAU.PS.1400.341**, whereas the experiments described in this study were conducted under the approved protocol **IR.SBMU.CRC.REC.1400.033**. This correction relates solely to the reporting of the ethics approval code and does not affect the conduct of the study or its scientific findings.

Third, clarification is provided regarding the euthanasia procedures described in the Methods section. Donor animals used for tumor tissue harvesting were euthanized by sodium pentobarbital overdose in accordance with the approved protocol, whereas recipient tumor-bearing mice at the experimental endpoint were euthanized by carbon dioxide inhalation followed by cervical dislocation under deep anesthesia. The published article did not clearly distinguish between these two categories of animals, resulting in an apparent inconsistency in the description of the euthanasia procedures. This correction clarifies the reporting of the experimental procedures only and does not reflect any deviation from the approved ethical protocol or the conduct of the study.

These corrections relate exclusively to reporting inaccuracies introduced during the final preparation and proofreading of the manuscript. The experiments were conducted using the correct primer sets and in full compliance with the approved ethical protocol. Accordingly, these reporting corrections do not affect the integrity of the experimental work, the validity of the reported data, the interpretation of the results, or the scientific conclusions of the article.

The authors would like to apologise for any inconvenience caused.

