# Supplementary material for: Nanochelate-based BCc1 delivery and its impact on key regulatory pathways in BALB/c breast cancer: An analysis of Beclin-1, ATG-4B, ATG-7, and mTOR expression
Source: Biochem Biophys Rep. 2026 Jan 6;45:102418. doi: 10.1016/j.bbrep.2025.102418 (PMC12808563; doi:10.1016/j.bbrep.2025.102418)

Size Distribution Report by Number

v2.2

Malvern

Sample Details

Sample Name: 40407-2078 1

SOP Name: Water 25 DTS 1060.sop

General Notes:

**File Name:**	1404.dts	**Dispersant Name:**	W/E
**Record Number:**	1065	**Dispersant RI:**	1.332
**Material RI:**	1.59	**Viscosity (cP):**	2.4323
**Material Absorbtion:**	0.010	**Measurement Date and Time:**	Tuesday, June 25, 2002 6:3...

System

**Temperature (°C):**	25.0	**Duration Used (s):**	3
**Count Rate (kcps):**	237.2	**Measurement Position (mm):**	5.50
**Cell Description:**	Clear disposable zeta cell	**Attenuator:**	9

Results

			**Size (d.nm):**	**% Number:**	**St Dev (d.n...**
**Z-Average (d.nm):**	73.17	**Peak 1:**	23.80	100.0	5.949
**PdI:**	0.872	**Peak 2:**	0.000	0.0	0.000
**Intercept:**	0.300	**Peak 3:**	0.000	0.0	0.000

Result quality : **Refer to quality report**

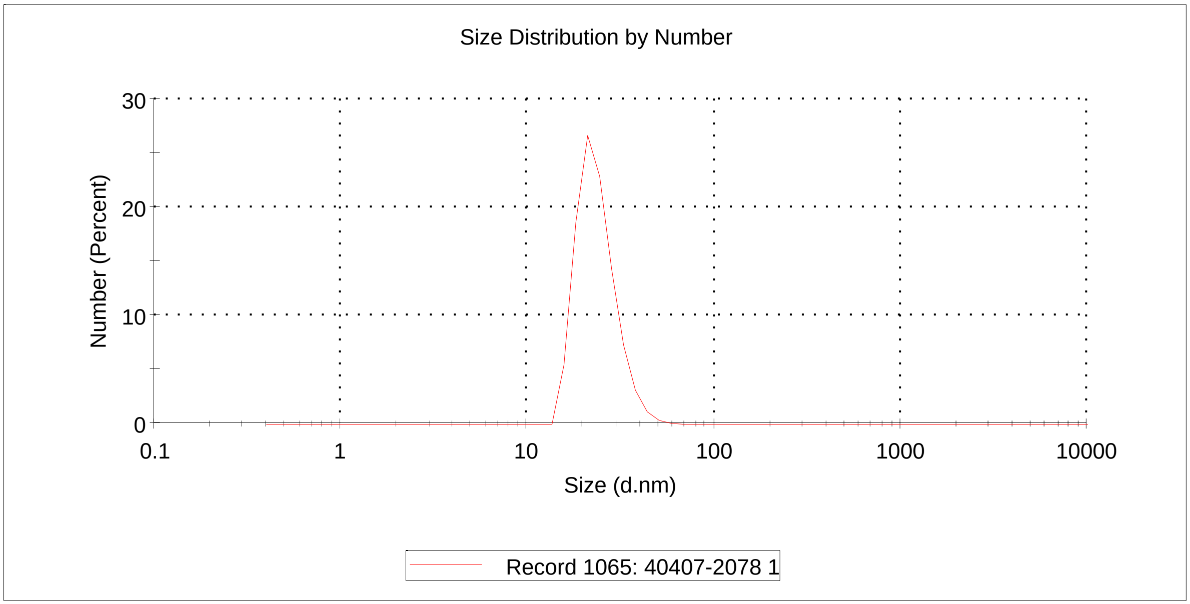

Supplement: Multimedia component 1 [file mmc1.pdf]
